# Colour preferences among individuals with common mental health disorders: a scoping review

**DOI:** 10.3389/fpsyg.2026.1832200

**Published:** 2026-07-22

**Authors:** Saara Krishnamurthy, Ruonan Shen, Frederick Sundram, Misha Vorobyev

**Affiliations:** 1 School of Optometry and Vision Sciences, Faculty of Medical and Health Sciences, University of Auckland, Auckland, New Zealand; 2 Department of Psychological Medicine, Faculty of Medical and Health Sciences, University of Auckland, Auckland, New Zealand

**Keywords:** colour preferences, diagnostic marker, mental health, mood and anxiety disorders, scoping review

## Abstract

**Introduction:**

Colours have been used as a complementary aid in the treatment of physical and mental disorders for centuries. There is substantial anecdotal evidence supporting the use of colours in therapeutic contexts, but scientific evidence remains limited. This review aimed to map existing literature on colour preferences among individuals with common mental health disorders, examine patterns across different diagnostic categories, and identify differences compared to healthy participants.

**Methods:**

A comprehensive literature search was conducted in four databases. This review followed the Preferred Reporting Items for Systematic reviews and Meta-Analysis extension for Scoping Reviews (PRISMA-ScR) and the Joanna Briggs Institute (JBI) guide for scoping reviews. Studies that assessed the favourite or preferred colour of individuals diagnosed with mental health disorders were reviewed. The methodological quality of the included studies was assessed using the Quality Assessment with Diverse Studies (QuADS) and Elliot’s guide for evaluating the quality of colour and psychological research.

**Results:**

A total of 16 studies met the inclusion criteria. Colour preferences were generally assessed using the Lüscher colour test, coloured cardboards, or digital colour representation. Distinct preferences for colours were observed across diagnostic categories—blue was preferred among those with depressive disorders, grey among those with anxiety-related disorders, and black among those with schizophrenia. Achromatic colours such as black or grey were not preferred by healthy participants. Healthy populations exhibited geographical variation in colour preferences, with European participants tending to prefer blue, while Asian participants preferred red and yellow. In contrast, no clear geographical patterns were observed among individuals with mental health disorders; preference patterns varied according to the diagnostic category.

**Conclusion:**

Colour preferences differed noticeably across various categories of common mental health disorders and from those of healthy individuals.

**Systematic review registration:**

The scoping review protocol was registered in the Open Science Framework and the URL: https://osf.io/khr8q.

## 1 Introduction

Nearly one in seven individuals around the world were living with a mental health disorder in 2019, and this prevalence increased by 26%–28% during the COVID-19 pandemic (World Health Organization, 2022). The development of fast and inexpensive methods of diagnosis and ancillary treatment protocols for common mental health disorders is essential to address the global mental health crisis. For centuries, colour therapy has been used in traditional medicine, with practitioners exposing patients to coloured lights applied to different body areas to promote calm or energise, influence mood, and support physical and mental health (Azeemi et al., 2019). Currently, colours are used as a complement in treating various disorders (Au and Assavarittirong, 2021; Darewych and Riedel Bowers, 2018; Fitria et al., 2022; Kim and Kang, 2013), and colour therapy is advertised on websites. However, scientific evidence demonstrating the effectiveness of colour therapy is absent (Azeemi and Raza, 2005). Since colours are associated with mood and emotions (Jonauskaite and Mohr, 2025), colours may potentially serve as markers of mental wellbeing, mental states, or mental health disorders (Max and Scott, 1969; Martín López and Fernández Díaz, 2022; Han and Lee, 2017).

Colour perception starts with light absorption by the three types of cone photoreceptors in the retina and can be described by three perceptual values (Wyszecki and Stiles, 2000). Perceptually, colour is characterised by hue, chroma, and lightness, enabling the communication of colours by ascribing values to each of these three dimensions. One of the most common systems for this kind is the Munsell atlas, which is widely used in colour vision research (Munsell, 1905; Newhall et al., 1943). Quantitative description of colour is based on the results of colour matching experiments, which form the basis of the Commission Internationale de l’Éclairage (CIE) XYZ and related colour systems (Commission Internationale de l’Éclairage, 2004). While the CIE XYZ colour system and the Munsell atlas system provide accurate representations of colour for the majority of people, these systems do not describe colours as they are seen by 8% of men and 0.5% of women with colour vision deficiency (Birch, 2012). Therefore, screening for colour deficiencies is essential in research.

People usually communicate their experience with colours using colour terms, such as ‘red’, ‘green’, and ‘blue’. These terms correspond to concepts of colours, which have different symbolic meanings in different cultures (Littlemore et al., 2023), and cannot be directly related to actual colours because they describe large colour categories with variable boundaries (Witzel, 2019). While the association between colour terms and emotions is largely defined by culturally specific symbolic meanings of the colour, the association between actual colours and emotions may tap into the unconscious mind that is less influenced by cultural conventions and therefore may be better suited as markers of mental wellbeing (Jonauskaite et al., 2020b; Wang et al., 2014; Littlemore et al., 2023).

Swiss psychotherapist Max Lüscher developed a colour test to identify personality traits and the current emotional state of an individual through the unconscious selection of colour preferences from a set of coloured plates (Max and Scott, 1969). While some studies supported the use of the Lüscher test for the diagnosis of psychological disorders (Turubarova et al., 2025; Stanzani Maserati et al., 2019; Rahn, 1976), others argued that the Lüscher test is an unreliable diagnostic tool (Donnelly, 1974; Picco and Dzindolet, 1994; French and Alexander, 1972). Unfortunately, the Lüscher test is not standardised; neither its CIE coordinates nor Munsell values have been documented. Therefore, it is difficult to compare results from different laboratories. Many studies on the psychological effects of colour do not accurately specify the colours that they use or do not control for colour deficiency. Nevertheless, such studies can serve as a baseline for exploring patterns of colour choice based on mood states. Improving their methodological rigour could help bridge the gap between colour science and psychological research.

When mapping colours to mental health assessment, colour preferences and colour-to-emotion associations can vary based on mood and behaviour. For example, people with mood disorders and anxiety-related disorders tend to prefer unsaturated, grey tones, while individuals with post-traumatic stress disorder (PTSD) avoid the colour red, as it potentially triggers underlying negative emotions (Bobic and Pavicevic Lukrecija, 2007). In contrast, healthy individuals usually dislike brown, black, white, or grey colours (Carruthers et al., 2010; Garvey and Luxenberg, 1987; Nolan et al., 1995; Ran et al., 2017; Sharma et al., 2012) and most often prefer yellow or bright colours (Carruthers et al., 2010). There were differences in the drawings of healthy participants and individuals with diverse psychological symptoms when observed under chromatic and achromatic conditions. However, the differences were more prominent in chromatic drawings, as the number of colours was less in individuals with diverse psychological symptoms, and black was the main colour in their drawings (Jue and Kwon, 2013).

There are reports of impaired motor and visual functions among individuals suffering from certain psychiatric conditions. Functional vision loss, reduced contrast sensitivity due to altered dopaminergic neurotransmission, and colour vision impairments are associated with depressive disorders, bipolar disorders, and schizophrenia (Salmela et al., 2021; Tao et al., 2024; Gupta et al., 2024; Tran et al., 2024; Barrick et al., 2002; Deisenhammer et al., 2022; Bubl et al., 2009). The increased colour discrimination thresholds, decreased colour sensitivity, and colour vision deficits have been observed in bipolar disorder, depressive disorders, and schizophrenia, respectively (Tran et al., 2024; Barrick et al., 2002). Reduced contrast gain at the retinal level has also been reported as a marker in depressed patients (Bubl et al., 2010). These changes at every level of processing information may explain the preference for unsaturated, dark-toned colours, and self-reports of a grey or colourless perception among individuals with mental health disorders.

This review aimed to determine whether individuals with mental health disorders differ in their colour preferences from healthy individuals and if colour preferences can be used as markers in the identification of mental health disorders. This review explored colour preferences using actual colours rather than colour terms, as colour terms are shaped by linguistic context or societal meanings, while responses to perceptual stimuli could reflect biological predispositions or sensory experiences with colour (Wang et al., 2014).

## 2 Methods

This review was registered in the Open Science Framework (https://osf.io/khr8q). This scoping review followed the Preferred Reporting Items for Systematic Reviews and Meta-Analyses extension for Scoping Reviews (PRISMA-ScR) (Tricco et al., 2018). This review adhered to the Joanna Briggs Institute (JBI) scoping review framework, and the population (adults with mental health disorders), concept (colour preferences), and context (different diagnostic categories with no restriction on gender, ethnicity, or geographic area) were used for the construction of the review (Pollock et al., 2023; Peters et al., 2024).

### 2.1 Identification of studies

#### 2.1.1 Inclusion and exclusion criteria

Studies were included in the review if (1) colour preferences were reported among adults aged more than 18 years with mental health disorders; (2) actual colours (not colour terms) were shown through printed paper/cardboards, wall paints, digital colours on the computer, or treatment in the form of coloured capsules; (3) at least three of the four unique hues were used; and (4) mental health disorders were identified based on validated methods such as the Diagnostic and Statistical Manual of Mental Disorders (DSM) or the ICD. The study selection was not restricted to a particular geographic location. Studies were excluded if (1) the studies only assessed colour preferences among healthy participants and not individuals with mental health disorders and (2) the colour choices were assessed through paintings/artworks of the participants.

#### 2.1.2 Search strategies and databases

The literature search for this review was conducted in three steps. The initial search was conducted in PubMed, OVID MEDLINE, Scopus, PsycINFO, and Google Scholar databases. The second search was conducted using appropriate keywords, Boolean operators, and subject headings in all the relevant databases. ProQuest and thesis searches were also used for the identification of grey literature. Finally, a manual search of the reference lists of the relevant literature was also performed. The literature search was restricted to English-language publications using English search terms and was conducted from database inception to December 2023. Studies that were not available in English translation were excluded. The search was conducted between May 2024 and August 2024 by two independent reviewers (SK and RS) and was reviewed again in May 2025. The detailed search strategy is provided in Supplementary material. The search outputs were exported to EndNote, and the selection of the studies, removal of duplicates, and screening of the literature were performed within EndNote. The study selection process is provided in a flowchart according to the Preferred Reporting Items for Systematic Reviews and Meta-Analyses extension for Scoping Reviews (PRISMA-ScR).

### 2.2 Data charting

The data were extracted in a pre-defined form that was specific to the current review. This extraction form was developed by one of the reviewers (SK) and was agreed upon by the other reviewers. After finalising the potentially eligible studies for review, the two independent reviewers evaluated the full text for inclusion in the current review, and reasons for exclusion were documented at each stage. The data extraction was carried out separately by the two reviewers, and the data were collated at the end to include all relevant information. Any discrepancy was resolved through discussions with the third reviewer (MV) until an agreement was reached.

A narrative synthesis of the review is reported, and the results are compiled according to the presentation and usage of colours, colour preferences among individuals with mental health disorders and healthy participants, and geographical or cultural differences in colour preferences.

### 2.3 Quality assessment

The quality appraisal was conducted to provide contextual insights into the methodological strengths and limitations of the included studies and was not used as a basis for study exclusion. The Quality Assessment with Diverse Studies (QuADS) and “Guidelines for a High-Quality Study on Color and Psychological Functioning,” as recommended by Elliot, were used to assess the methodological quality of the reviewed studies (Harrison et al., 2021; Elliot, 2019). The first tool consists of 13 items rated on a 3-point scale, with 0 representing no mention at all and 3 representing complete information, and the second tool consists of five items evaluated as one if they were controlled and zero if they were not controlled (Table 1). Raw scores and percentages were reported directly, without assigning quality rankings, in accordance with the recommendations of the selected appraisal tools. Extraction of data and quality assessments were conducted separately by the two independent reviewers, and the inter-rater reliability was assessed using intraclass correlation (ICC) with a 95% confidence interval.

**Table 1:** Quality assessment of the reviewed studies by the reviewers.

S no	Reviewed studies	Q1.1		Q1.2		Q1.3		Q1.4		Q1.5		Q1.6		Q1.7		Q1.8		Q1.9		Q1.10		Q1.11		Q1.12		Q1.13		Score	Q2.1		Q2.2		Q2.3		Q2.4		Q2.5		Score
		SK	RS	SK	RS	SK	RS	SK	RS	SK	RS	SK	RS	SK	RS	SK	RS	SK	RS	SK	RS	SK	RS	SK	RS	SK	RS	Total (%)	SK	RS	SK	RS	SK	RS	SK	RS	SK	RS	Total (%)
1	Carruthers et al. (2010)	2	3	3	2	3	3	2	2	3	2	2	2	3	2	3	2	2	2	3	3	3	3	0	2	1	2	76.9	0	1	0	1	0	1	1	0	1	0	40
2	Garvey and Luxenberg (1987)	2	3	2	1	2	2	2	2	2	2	2	2	2	2	2	2	2	2	2	2	2	2	0	2	2	2	66.6	0	1	0	0	0	0	0	0	0	0	0
3	Korkmaz et al. (2016)	3	1	2	2	2	2	2	2	2	2	2	3	2	3	2	2	2	3	2	2	2	3	0	1	2	1	69.2	0	0	0	0	0	1	0	0	0	0	0
4	Kuloğlu et al. (2002)	3	2	3	1	2	3	2	2	2	2	2	2	2	2	3	3	3	3	2	2	2	3	0	2	1	1	71.7	0	1	0	0	0	1	0	0	0	0	0
5	Nolan et al. (1995)	2	2	2	1	2	2	2	2	2	2	2	2	2	2	2	2	2	2	0	1	0	1	0	0	0	0	48.7	1	1	0	0	0	1	0	0	0	0	20
6	Ran et al. (2017)	1	2	2	3	3	3	3	3	3	2	2	2	2	2	3	2	3	2	2	2	2	3	3	1	2	2	74.3	1	1	1	0	0	1	0	0	0	0	40
7	Ross et al. (2010)	3	3	2	1	3	2	3	3	3	2	2	3	3	3	3	3	3	2	0	3	1	3	2	0	1	1	74.3	1	1	1	1	0	0	1	1	1	1	80
8	Schapira et al. (1970)	2	2	2	2	2	2	2	2	2	2	2	2	2	2	2	2	2	2	2	1	2	2	2	1	1	2	61.5	1	1	1	1	0	0	0	0	0	0	40
9	Sharma et al. (2012)	2	3	2	1	1	2	2	2	2	2	2	1	2	2	1	2	1	2	0	1	1	1	0	0	2	2	53.8	0	0	0	0	0	0	0	0	0	0	0
10	Hettiarachchi and Perera (2021)	2	2	2	3	2	3	2	3	2	1	3	1	2	2	1	1	2	1	1	2	1	1	0	2	1	1	58.9	0	0	0	0	0	1	1	1	0	0	20
11	Bobic and Pavicevic Lukrecija (2007)	3	2	2	2	2	3	3	2	3	2	3	2	3	2	1	2	2	3	0	2	2	1	1	2	2	1	66.6	0	1	1	1	0	1	0	0	0	0	20
12	Tao et al. (2015)	2	2	2	2	2	2	3	2	2	2	2	2	2	2	2	2	2	2	3	1	2	2	1	2	2	2	64.1	0	1	1	1	0	1	1	1	1	0	60
13	Fernando et al. (1992)	2	2	2	1	2	2	2	2	1	2	2	1	2	1	1	3	1	1	0	2	1	1	3	2	1	2	56.4	0	1	0	0	0	0	0	0	0	0	0
14	Cernovsky and Fernando (1988)	2	2	1	1	2	1	2	2	1	1	2	1	2	1	1	2	1	1	0	2	1	1	0	0	1	2	43.5	0	1	0	0	0	0	0	0	0	0	0
15	Cernovsky et al. (1997)	2	3	2	1	2	2	2	2	2	2	3	2	3	1	1	2	1	1	1	2	1	1	2	2	2	2	58.9	0	1	0	0	0	0	0	0	0	0	0
16	Holmes et al. (1985)	2	1	2	1	2	2	2	2	2	2	2	2	2	1	2	2	1	1	1	2	2	1	2	2	1	2	53.8	0	1	0	0	0	0	0	0	0	0	0

Q1.1: The theoretical concepts supporting the research, Q1.2: clear statement of aims, Q1.3: research setting, Q1.4: target population, Q1.5: appropriate sampling techniques, Q1.6: rationale for choice of data collection tool, Q1.7: format and content of data collection tool, Q1.8: description of data collection tool, Q1.9: recruitment data provided, Q1.10: justification of analytic method selected, Q1.11: the appropriate method of analysis, Q1.12: inclusion of appropriate stakeholders in research, and Q1.13: critical discussion of strengths and weaknesses. Q2.1: Participant naiveté and confidentiality, Q2.2: assessment of and the omission of participants with colour vision deficiency, Q2.3: sample size estimation, Q2.4: colour specification and control, and Q2.5: background colour and illumination.

## 3 Results

### 3.1 Selection of literature

**Figure 1:**
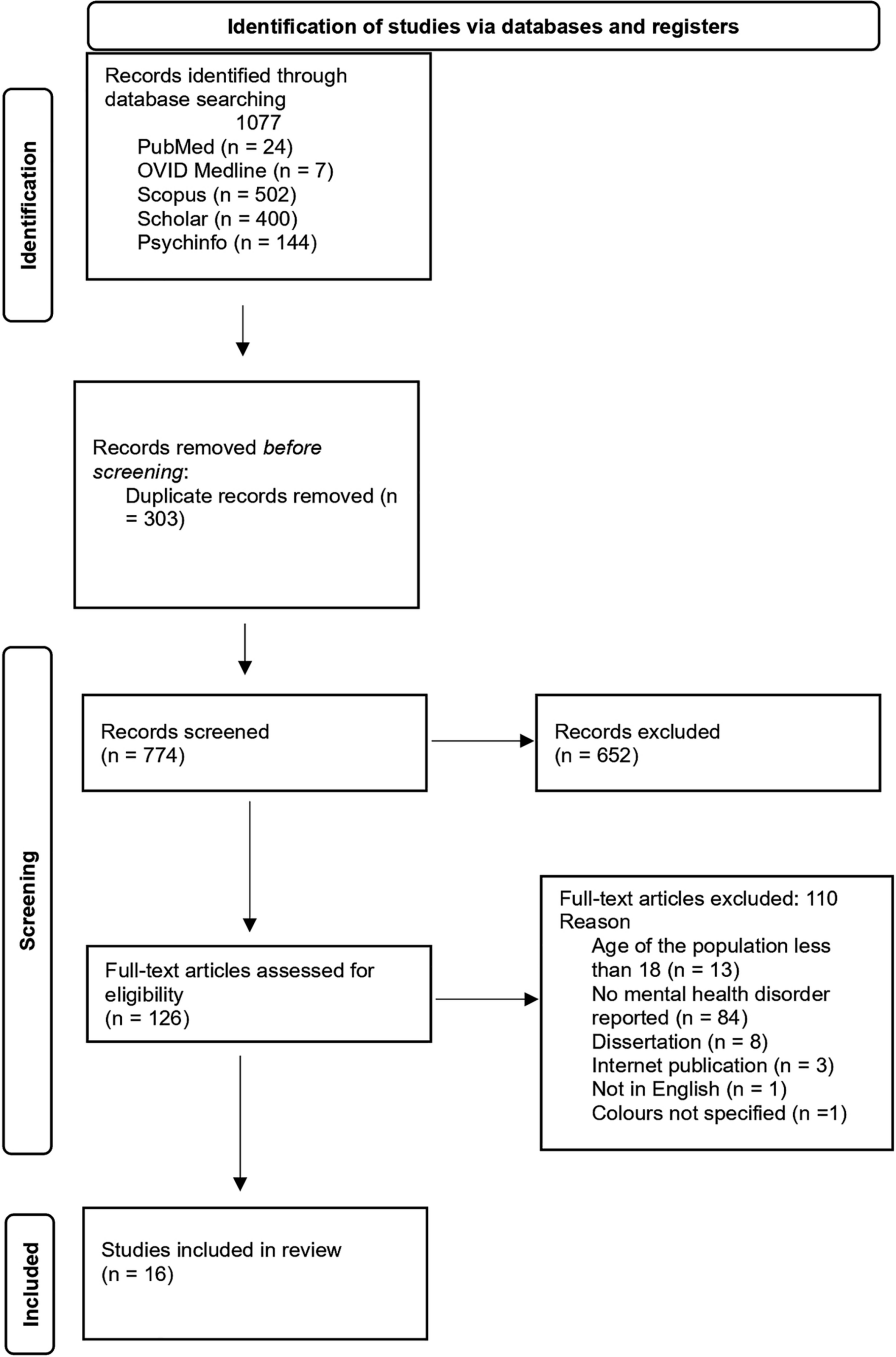
Flowchart of the search strategy and study selection processes. Adapted from Page et al. (2021).

The initial literature search in the databases resulted in 1,077 articles. After removing duplicates, there were 774 articles for title and abstract screening, and the full texts of 126 articles were screened. This review included 16 published works, and the data were extracted by two independent reviewers. Figure 1 represents the flowchart of the study review process. There were 4,239 participants in the 16 included studies, among whom 35.98% (*n* = 1,525) were healthy volunteers and 64.02% (*n* = 2,714) were diagnosed with common mental health disorders. Depressive disorders (15.4%) were the most commonly studied, followed by anxiety and related disorders (10%), post-traumatic stress disorder (4.4%), schizophrenia (3.5%), and somatoform disorders (2%). Two of the studies did not classify participants (n = 1,212, 28.59%) into specific disorders. Table 2 provides a summary of this review.

**Table 2:** Summary of the results from the review of 16 studies.

Sno	Title	Mental health disorder category	Brief methodology	Presentation of colours	Number of colours used	Identification of mental health disorder	Preferred colour—mental health disorder (in order)	Preferred colour—healthy participant	Geography	Reports before and after therapy Methods and results
1	Carruthers et al. (2010)	Depressive and anxiety-related disorders	Favourite colour and mood colour were assessed through a questionnaire that included an assessment of depression and anxiety, as well as the colour preferences.	Colour wheel based on The Munsell system was developed and printed on paper Hues—red, yellow, orange, green, blue, purple Four shades of each of the above colours Pink and brown Two achromatic colours—black and white	38	HAD scale	Blue Grey	Blue	Europe	No
2	Garvey and Luxenberg (1987)	Depressive disorder	Colour preferences were assessed among patients referred to a psychiatric clinic and were compared with those of healthy controls	Abstract colours Lüscher colour test	8	Hamilton depression scores Patients referred from the psychiatric outpatient department Controls were staff from the hospital	No difference	Yellow	America	No
3	Korkmaz et al. (2016)	Depressive and anxiety-related disorders	Participants were asked the first colour that comes to mind and their favourite colour using coloured cardboards	Abstract colours Coloured cardboards	8	Beck anxiety depression inventory	Blue Black Grey	Not assessed	Europe	No
4	Kuloğlu et al. (2002)	Depressive and anxiety-related disorders	Participants were asked to select a preferred colour from a set of colours presented on a cardboard	Abstract colours 4 cm^2^-coloured cardboards	8	DSM 4	Green	Not assessed	Europe	No
5	Nolan et al. (1995)	Depressive disorder	Participants were given a demographic questionnaire, followed by printed colours and the Beck Depression Inventory	Abstract colours Colours printed on the paper, along with a questionnaire	7	BDI	Black	Other colours except black or brown	America	No
6	Ran et al. (2017)	Depressive and anxiety-related disorders	Colour preferences for abstract colour and environmental colours were assessed. For abstract colours, pairs of printed colours were presented, and for environmental colours, digital stimuli of different hospital ward walls were presented.	Abstract colours—coloured cards Environmental colours—hospital wards with different wall colours presented digitally	9	HAMA and HAMD	Blue	Red Blue Yellow	Asia	Antidepressant drug therapy No significant difference before and after therapy
7	Ross et al. (2010)	Depressive disorder	A pilot study that evaluated the effectiveness of colour therapy. Change in colour preferences before and after light therapy was assessed	Audio-Bio Colour system—digitally administered colour preference test 12 rows, 8 colours in squares presented in random order Each colour is presented 8 times Participants are instructed to discard 4 colours in each row	12	DSM 4 and for the level of depression PHQ9	Blue	Not assessed	America	A weekly light session/colour routine was given as therapy. Different coloured lights were projected on the participant’s body Before therapy—blue After therapy—green and red
8	Schapira et al. (1970)	Anxiety-related disorder	Antidepressant drugs were administered in tablets of three different colours. The effectiveness of using different coloured tablets was assessed, thereby assessing the role of colours in the treatment of mental health disorders	Antidepressant drugs were given in the form of different coloured tablets	3	Clinically identified patients with anxiety during treatment A self-rated scale was used that was developed by Lader and Wing	No difference	Not assessed	Europe	Antidepressant drug therapy No significant difference before and after therapy
9	Sharma et al. (2012)	Depressive and anxiety-related disorders	The Lüscher colour test was used to identify the colour preferences among individuals with anxiety- and mood-related disorders	Abstract colours Lüscher colour test	8	ICD depression criteria	Grey	Blue Dislike black	Asia	No
10	Hettiarachchi and Perera (2021)	Depression, anxiety, and stress	Individuals with mobility impairments were assessed for mood- and anxiety-related disorders. They were exposed to different coloured wall panels in the hospital ward. The depression and anxiety ratings before and after exposure to different coloured wall panels were assessed	Environmental colours—wall colour	3	DASS 21S	Green	Not assessed	Asia	The wall panel was installed, and the participants were exposed to different wall colours. Depression and anxiety improved in green colour Yellow caused mild anxiety.
11	Bobic and Pavicevic Lukrecija (2007)	Post-traumatic stress disorder (PTSD)	The colour preferences of prisoners of war were assessed to understand the association between PTSD and colour preferences	Abstract colours Lüscher colour test	8	DSM IV Psychodiagnostics testing and interviews	Green Violet	Not assessed	Asia	No
12	Tao et al. (2015)	Schizophrenia	Colour preferences of individuals with schizophrenia were compared with those of healthy volunteers	Abstract colours Colours presented on a monitor with a grey background colour	11	Schizophrenia diagnosed using ICD 10, followed by the ZKPQ test to assess personality traits	Black Brown	Green Yellow	Asia	No
13	Fernando et al. (1992)	Bipolar and schizophrenia	Colour preferences among individuals with mania, schizophrenia, and healthy volunteers were assessed to understand the role of colour preferences as a diagnostic sign	Abstract colours Lüscher colour test	8	DSM III R ICD 9	No difference	No difference	America	No
14	Cernovsky and Fernando (1988)	Schizophrenia	Colour preferences among individuals with schizophrenia and healthy volunteers	Abstract colours Lüscher colour test	8	DSM III and ICD 9	No difference	No difference	America	No
15	Cernovsky et al. (1997)	Schizophrenia	Colour preferences of individuals with borderline personality disorders and healthy individuals were assessed	Abstract colours Lüscher colour test	8	DSM III R	Black Red	Grey brown	America	No
16	Holmes et al. (1985)	NA	The association between colour preferences and psychiatric disorders was assessed	Abstract colours Lüscher colour test	8	DSM III	Yellow Red Blue	Not assessed	America	No

### 3.2 Classification of mental health disorders

Mental health disorders were classified based on validated scales or clinical assessment. The Diagnostic and Statistical Manual of Mental Disorders (DSM) was commonly used for the classification of common mental health disorders, and the version varied based on the year of administration. The Beck Depression Inventory (BDI), Beck Anxiety Inventory (BAI), the Barratt Impulsiveness Scale (BIS), the Hamilton Depression Rating Scale/Hamilton Anxiety Rating Scale (HAMD and HAMA), the Hospital Anxiety and Depression Scale (HADS), and the Depression, Anxiety, and Stress Scale (DASS) were used as measures (Table 2).

### 3.3 Usage of colours in the assessment of colour preferences

The number of colours used in the assessment of colour preferences ranged between 3 and 38, while the majority of studies used the Lüscher colour test. Korkmaz et al. (2016) and Kuloğlu et al. (2002) used the same colours as those used in the Lüscher test but replaced violet with pink to understand the association between colour and emotion (Korkmaz et al., 2016; Kuloğlu et al., 2002). However, there was no explanation for using pink instead of violet. Hettiarachchi and Hettiarachchi and Perera (2021) and Schapira et al. (1970) used only three of the four unique hues (red, green, yellow, and blue) (Schapira et al., 1970; Hettiarachchi and Perera, 2021). Achromatic colours (black and white) were used in 14 of the 16 studies (Table 2).

As the representation of colours plays a major role in understanding the relationship between sensory perception of colour and emotional response, Carruthers et al. (2010) developed a colour wheel based on the Munsell colour system with six primary colours (orange in addition to the five principal Munsell colours—red, green, yellow, blue, and purple), pink, and brown (Carruthers et al., 2010). These eight primary colours, along with achromatic colours (black, white, and grey), were presented in four different saturation levels/shades. This provided the participants a wider range of colour choices and may also help in understanding the connotations of the dark and light shades of the same colour.

### 3.4 Colour preferences among healthy individuals

Healthy participants in the included studies (Carruthers et al., 2010; Garvey and Luxenberg, 1987; Nolan et al., 1995; Ran et al., 2017; Sharma et al., 2012; Tao et al., 2015; Fernando et al., 1992; Cernovsky and Fernando, 1988; Cernovsky et al., 1997) reported varied colour preferences, and yellow was the most preferred colour, followed by green, red, and blue. None of the reviewed studies reported healthy participants preferring black, grey, or white.

### 3.5 Colour preferences among individuals with mental health disorders

Studies reported either the frequency of the preferred colour or favourite colour (Carruthers et al., 2010; Korkmaz et al., 2016; Kuloğlu et al., 2002; Nolan et al., 1995; Sharma et al., 2012; Hettiarachchi and Perera, 2021; Bobic and Pavicevic Lukrecija, 2007; Holmes et al., 1985) or ranked the preference of colour from most appealing to least appealing (Garvey and Luxenberg, 1987; Ross et al., 2010; Fernando et al., 1992; Cernovsky et al., 1997; Cernovsky and Fernando, 1988; Ran et al., 2017; Tao et al., 2015; Schapira et al., 1970) (Table 2). The results of colour preferences are compiled as the colour preferences for abstract colours, environmental colours, and the change in colour preferences after therapy (Table 2). Abstract colours refer to the representation of colours on paper, cardboard, or digital screens and environmental colours refer to the representation of colour in indoor settings such as room colour or wall colour.

When participants were instructed to pick a colour from printed sheets or digital materials without any specific instruction, blue was the preferred colour, followed by red, green, and grey. Participants with depressive disorders preferred blue and grey, while violet and brown were their least preferred colours (Carruthers et al., 2010; Korkmaz et al., 2016; Ran et al., 2017; Ross et al., 2010; Sharma et al., 2012). Participants with anxiety and related disorders preferred grey but did not prefer brown or yellow, while participants with post-traumatic stress disorder preferred violet and green equally, with the least preference given to brown, red, and grey (Bobic and Pavicevic Lukrecija, 2007; Hettiarachchi and Perera, 2021; Kuloğlu et al., 2002).

When participants were asked to rank colours in order of their liking, blue was ranked first, followed by green among participants with depressive disorders, while those diagnosed with schizophrenia preferred black more than other colours (Cernovsky et al., 1997; Tao et al., 2015; Garvey and Luxenberg, 1987; Ran et al., 2017). Healthy volunteers and participants with mental health disorders ranked the colours similarly, with no significant differences in the order of colour ranking (Fernando et al., 1992; Cernovsky and Fernando, 1988).

The preferences for colour for indoor environments, such as hospitals, classrooms, or indoor decor, were evaluated among participants with schizophrenia and depressive disorders. Participants with schizophrenia preferred black and red for the living room decor but did not prefer grey (Cernovsky et al., 1997). Individuals with depressive disorders preferred blue for the classroom environment and green for the hospital environment but did not prefer yellow, as it induced anxiety (Hettiarachchi and Perera, 2021). Among those with depressive disorders, purple ranked second among the preferred colours for indoor environments, while yellow, orange, black, and grey were the least preferred colours (Ran et al., 2017).

### 3.6 Change in colour preferences after therapy

The colour preferences were assessed before and after therapeutic interventions using two methods: therapy using antidepressants (Ran et al., 2017; Schapira et al., 1970) and exposure to colours as a therapy (Ross et al., 2010; Hettiarachchi and Perera, 2021) (Table 2). Antidepressant drugs were administered as coloured capsules, and the mood status of the participants was assessed using a self-rating scale and physician assessment (Schapira et al., 1970). No statistically significant differences were observed between different coloured capsules, but a trend towards improved mood with yellow-coloured capsule was observed (Schapira et al., 1970). In addition, during antidepressant therapy, preference for grey decreased after therapy, and preference for yellow increased (Ran et al., 2017). Participants were exposed to colours on a digital screen as therapy, or the room colours were altered to understand the preferences of colour before and after therapy. The colour preferences after exposure to different colours resulted in increased preferences for green and red and a decreased preference for blue (Hettiarachchi and Perera, 2021; Ross et al., 2010).

### 3.7 Geographical and cultural preferences

The studies were conducted across Asian (*n* = 5), American (*n* = 7), and European (*n* = 4) regions, with nine studies reporting colour preferences among healthy participants (Table 2). A distinct pattern was observed among healthy volunteers: Europeans preferred blue, Asians preferred red and yellow, and healthy Americans exhibited more varied preferences. Individuals diagnosed with mental health disorders did not report a consistent region-specific pattern of colour preferences (Table 2). Instead, colour preferences appeared to be influenced by the diagnostic category. Participants with depressive disorders and anxiety-related disorders preferred blue and green, whereas those diagnosed with schizophrenia preferred black.

Given that both healthy European participants and individuals with depressive disorders preferred blue, this pattern was examined further within the reviewed studies. Among the included studies, only one European study directly compared healthy volunteers with individuals with depressive disorders using blue hues of varying brightness (Carruthers et al., 2010). In this study, healthy volunteers preferred brighter shades of blue, while those with depressive disorders preferred darker shades of blue. The remaining studies did not include hues with varying brightness, limiting broader interpretation. These findings suggest that, while geographical trends may influence general colour preference in healthy populations, diagnostic factors may play a more substantial role in shaping colour preferences within clinical populations.

### 3.8 Quality of the studies

The inter-rater agreement is represented using the intraclass correlation coefficient, with a 95% confidence interval. The scores for the quality of studies are presented as a percentage by calculating the number of reported criteria divided by the total number (39 for QuADS and 5 for the guidelines by Elliot). For example, when a study reported the experimental setup, including background and illumination, question number five would be rated 1, if not zero. Using the QuADS tool, the score ranged between 41.03 and 79.49%, with an average score of 61.7% ± 11.5, and the intraclass correlation coefficient was found to be 0.92 (95% confidence interval: 0.80—0.97). The score for the tool developed by Elliot, specifically for colour psychology studies, ranged between 0 and 80%, and the majority of the studies had a score of 0 (Table 1). The intraclass correlation coefficient for quality assessment was found to be 0.91 (95% confidence interval: 0.71—0.97). Table 1 provides the quality assessment conducted by the reviewers.

## 4 Discussion

Colour preferences among individuals with mental disorders are distinct from those of healthy individuals and depend on the type of mental health disorder. There is a widespread interest in the topic of the relationship between colours and mental health, with many informal reports and anecdotal evidence available on the Internet, but there are only 16 published studies that examined the colour preferences among individuals with mental health disorders.

The general methodological quality assessment showed that overall quality was moderate. The questions that assess the analytical methods adopted and the integration of stakeholders in designing the research and data collection were graded the lowest. The quality assessment of colour psychology studies also showed that many studies did not account for the standards recommended in colour science, including diverse samples and cross-cultural validation. The majority of the studies in the review did not control for colour vision deficiency. Although no significant association between colour vision deficiency and colour–emotion association has been reported, individuals without colour vision deficiency were more consistent in associating colours with emotions than those with colour vision deficiency (Jonauskaite et al., 2021). Lack of control for colour vision deficiency can contribute to inconsistencies in the reporting of colour preferences.

Another important methodological consideration relates to the presentation and specification of colour dimensions. The reviewed studies used colour stimuli that lacked quantitative specifications, such as the Lüscher Colour Test, coloured card samples, or wall colours. Colours were described with colour names such as ‘red’, ‘green’, and ‘blue’, without specification of hue, lightness, or chroma, and stimulus presentation conditions, such as lighting and screen calibration, were not reported. This absence of colorimetric specification makes it impossible to verify whether colours labelled identically across studies were perceptually equivalent. Future research should adopt precise stimulus presentation by reporting standardised colorimetric coordinates and lighting conditions to improve reproducibility and comparability across studies.

Colour preferences and colour–emotion associations generally depend on culture, gender, and ethnicity (Taylor et al., 2013; Jonauskaite et al., 2020a). European men prefer blue/green colours over red, while European women prefer shades of red (Al-Rasheed, 2015). In China, both men and women prefer red, as it is a symbol of luck (Cheng et al., 2009; Luo, 2022; Wang et al., 2014). Indian women prefer orange to yellow hues, while British women prefer lavender hues (Bonnardel et al., 2012). Colour preferences among healthy participants show a consistent pattern when they are from geographically close regions (Jonauskaite et al., 2020a). However, the colour preferences among individuals with mental health disorders showed no region-specific pattern but were distinct for each category of mental health disorder (Table 2).

Individuals with depressive disorders, anxiety disorders, and schizophrenia preferred blue, grey, and black colours, respectively (Table 2). The colour preferences after treatment changed to colours that were similar to those preferred by healthy individuals (Ran et al., 2017; Schapira et al., 1970; Ross et al., 2010; Hettiarachchi and Hettiarachchi and Perera, 2021). The mechanism of these preferences is still not clearly understood. For example, blue is reported to have both positive (calming and pleasant) and negative (depressing and sad) valence. We do not know whether individuals with depressive disorders prefer blue because they find it calming or because it is associated with feelings of depression. Individuals with depressive disorders generally show sad mood, insomnia, decreased energy levels, decreased psychomotor activity (Sekhon and Gupta, 2024), and their tendency to prefer calmness is substantial, as explained by the emotional valence theory (Palmer and Schloss, 2010; Taylor and Franklin, 2012). Preference for black among those with schizophrenia may relate to the colour’s widely recognised symbolic association (black mood, fear, unknown, and absence) and the emotional state of the individual (neuroticism) (Tao et al., 2015). One study has reported the colour preferences of prisoners of war with post-traumatic stress disorder, and they did not prefer the colour red as it triggered past traumatic events (Bobic and Pavicevic Lukrecija, 2007). Exploring colour preferences could be a useful tool in the identification of the disorder, planning of treatment approaches, and monitoring of disease progression.

This review followed established guidelines for conducting a scoping review and included studies assessing colour preferences among individuals with mental health disorders. The included studies were heterogeneous in their experimental designs and diagnostic criteria for mental health conditions, which limited the direct comparability and generalisability of the findings. Although the search strategy included major databases and grey literature sources, it was conducted using English-language search terms only. This may have introduced selection bias and limited the inclusion of relevant studies published in other languages and not translated to English, thereby narrowing the breadth of the evidence base.

## 5 Conclusion

Colour preferences vary systematically across mental health disorders and differ from those of healthy individuals. Individuals with mood disorders, anxiety disorders, PTSD, and schizophrenia tend to prefer darker, unsaturated colours, whereas healthy participants favour brighter hues. These patterns should be interpreted with caution given the moderate methodological quality and heterogeneity of the included studies, including a lack of standardised colour specification. Colour has the potential to be used as a complementary marker in the diagnosis and monitoring of mental health disorders. However, a necessary step in this direction is using stimuli that are colorimetrically characterised.

## Data Availability

The original contributions presented in the study are included in the article/Supplementary material, further inquiries can be directed to the corresponding author.
