# Supplementary material for: Colour preferences among individuals with common mental health disorders: a scoping review
Source: Front Psychol. 2026 Jul 22;17:1832200. doi: 10.3389/fpsyg.2026.1832200 (PMC13437323; doi:10.3389/fpsyg.2026.1832200)
Supplement: Supplementary file 1 [file Table_1.DOCX]

Supplementary material 1

Detailed search strategy

As there were many works on zebra fish during the initial search in the PubMed, the term zebra fish and animal was explicitly excluded from the keywords. The following keywords were used in the PubMed database.

(“Color preference” OR “Colour preference”) AND (“Mood” OR “emotions” OR “mental health” OR “depression” OR “anxiety” OR “major depressive disorder” OR “affective disorder” OR “schizoph**” OR “bipolar”) NOT (“animal” OR “zebrafish”)

(“Color choice” OR “Colour choice”) AND (“Mood” OR “emotions” OR “mental health” OR “depression” OR “anxiety” OR “major depressive disorder” OR “affective disorder” OR “schizoph**” OR “bipolar”) NOT (“animal” OR “zebrafish”)

("Color choice" OR "Color preference" OR "Colour choice" OR "Colour preference") AND (“emotions” OR “mood” OR “schizoph**” OR “bipolar”) AND ("consumer" OR "customer" OR "purchase" OR "online" OR "store" OR "environment")

In the other databases the following keywords were used.

(“Color preference” OR “Colour preference”) AND (“Mood” OR “emotions” OR “mental health” OR “depression” OR “anxiety” OR “major depressive disorder” OR “affective disorder” OR “schizoph**” OR “bipolar”) AND (“people” OR “human” OR “individuals” OR “adults”)

(“Color choice” OR “Colour choice”) AND (“Mood” OR “emotions” OR “mental health” OR “depression” OR “anxiety” OR “major depressive disorder” OR “affective disorder” OR “schizoph**” OR “bipolar”) AND (“people” OR “human” OR “individuals” OR “adults”)

("Color choice" OR "Color preference" OR "Colour choice" OR "Colour preference") AND (“emotions” OR “mood”) AND ("consumer" OR "customer" OR "purchase" OR "online" OR "store" OR "environment")
